# Asphaltenes from Heavy Crude Oil as Ultraviolet Stabilizers against Polypropylene Aging

**DOI:** 10.3390/polym15214313

**Published:** 2023-11-03

**Authors:** Viktoria Y. Melekhina, Anna V. Vlasova, Sergey O. Ilyin

**Affiliations:** A.V. Topchiev Institute of Petrochemical Synthesis, Russian Academy of Sciences, 29 Leninsky Prospect, 119991 Moscow, Russia

**Keywords:** polypropylene, asphaltenes, UV aging, UV stabilizers, polymer destruction, polymer composites, UV resistant composites, rheology, crystallinity, strength

## Abstract

The destruction of polymers under the influence of ultraviolet (UV) radiation is the cause of their aging and deterioration of strength properties. Asphaltenes are low-value waste products after the refining and deasphalting of heavy crude oil, which absorb UV radiation well. Asphaltenes require rational utilization, which suggests their use as UV stabilizing agents for polymers. In this work, asphaltenes were used to prevent UV aging of polypropylene (PP) by adding them in a mass fraction from 5% to 30% within an asphaltene/PP composite material. Rheometry, calorimetry, X-ray diffraction analysis, and tensile strength of PP films containing asphaltenes were performed before and after their intense UV irradiation for accelerated aging. Asphaltenes slightly reduce the viscosity, crystallinity, and mechanical strength of the initial PP due to their plasticizing effect. However, this deterioration in properties is more than compensated when studying UV-aged samples. Intense UV aging causes multiple catastrophic drops in the viscosity and strength of pure PP with the preservation of crystallinity due to the break of polymer chains and a decrease in molecular weight by approximately eight times. Asphaltenes suppress the destruction of PP, which is expressed in a significantly smaller decline in its viscosity and strength due to UV aging. The most optimal content of asphaltenes is 20%, which suppresses UV destruction by six times and best preserves the strength properties of PP.

## 1. Introduction

Ultraviolet (UV) radiation is well known for its damaging effects, including on polymers [1,2,3]. The consequences include changes in the operational properties and the material surface, such as color fading, reduced product strength, increased fragility, and a violation of other mechanical properties [4,5]. Special modifying additives (UV absorbers) are added to the composition of polymers to improve their resistance to ultraviolet rays [6,7,8]. These stabilizers absorb not only UV rays but also free radicals [9], increasing the thermal and thermal-oxidative resistance of the modified material [10,11,12].

Initially, mainly Ni-containing compounds were used as UV absorbers, for example, to stabilize low-density polyethylene films used in agriculture and horticulture [13,14]. Currently, inorganic oxides, such as oxides of zinc, cerium, silicon, and titanium, are employed more often to obtain UV protective coatings in the form of a pure protective oxide layer or dispersed pigments [15,16,17,18,19,20]. In addition, there are wide applications of organic UV stabilizers whose molecules most often include a phenol group and intramolecular O-H-O or O-H-N bridges, which play a significant role in the dissipation of absorbed energy [21,22,23,24]. Non-phenolic organic UV absorbers, such as oxanilides and cyanoacrylates, are also in use, exhibiting high photochemical stability [25,26]. Synthetic organic UV stabilizers have complex molecular structures, resulting in relatively high production costs due to the multi-stage synthesis. This drives the search for new and cheaper alternatives to prevent polymer UV aging.

A modern approach to modifying polymers is the growth in the use of natural and industrial waste products [27,28,29,30], which include asphaltenes [31,32]. The molecules of asphaltenes, which are present in heavy crude oil [33], contribute to its high viscosity [34,35] and remain even after its deasphalting [36,37], which is performed to facilitate oil pipeline transportation [38,39,40]. Rational utilization of residual asphaltenes is a significant environmental problem, which is solved by using asphaltenes in the production of emulsions [41,42,43], carbon fibers [44,45,46,47], and bitumen binders [48,49]. Furthermore, asphaltenes are an inexpensive filler used to improve the operational properties and reduce the cost of polymer materials [50], such as epoxy plastics [51,52,53,54], polyethylene [55,56,57,58,59], polypropylene [60], polyisobutylene [61], poly(methyl methacrylate) [62], polystyrene [63,64], and its copolymers [65,66,67]. Asphaltene-containing composite plastics can be used in many industries, except for products that have direct contact with human skin or food due to the potential carcinogenicity of asphaltenes [68,69].

In recent years, asphaltenes have been used as stabilizers against thermal oxidative degradation [70], as they suppress the adverse action of free radicals. Moreover, asphaltenes can act as UV absorbers [71], which potentially may be utilized for ultraviolet protection [61] and anti-aging of polymers [72]. However, none of the existing studies have explored the possibility of using asphaltenes as UV stabilizers for preventing polymer aging and the resulting deterioration of their mechanical properties.

Polypropylene (PP) is one of the most common polymers in the plastics industry today and is highly susceptible to the harmful effects of ultraviolet radiation [73,74,75,76], explaining the choice of PP as the model polymer matrix for this pioneering study of asphaltene UV absorbing ability. It aims to obtain and investigate composite materials based on polypropylene and asphaltenes and to evaluate the effectiveness of asphaltenes as stabilizers against UV destruction.

## 2. Materials and Methods

### 2.1. Materials

Isotactic polypropylene (PP) Montell 6100 (LyondellBasell, Hoofddorp, The Netherlands) has a melting temperature of 166.6 °C, a molecular weight of 2.3 × 10^5^ g/mol, and a melt flow index of 12 g/10 min (load: 2.16 kg, temperature: 230 °C). Asphaltenes were isolated from heavy crude oil from the Ashalchinskoye oil field (Tatarstan, Russia) using hexamethyldisiloxane as a nonsolvent (Ecos-1, Moscow, Russia) and a nonsolvent/oil volume ratio of 15:1. The initial heavy crude oil has a density of 0.962 g/cm^3^ and a viscosity of 4.25 Pa·s at 20 °C and consists of 7.5% heptane-insoluble asphaltenes, 16.2% acid (benzene/ethanol soluble) resins, 7.6% neutral (benzene-soluble) resins, 36.0% polycyclic aromatics, 5.5% bicyclic aromatics, 4.1% monocyclic aromatics, and 23.1% saturates. Its mass C/H/N/S composition was 82.6/11.2/0.29/3.9 [48]. The asphaltene isolation procedure has been described in detail previously [77]. The resulting asphaltene product consists of 19.3% heptane-insoluble asphaltenes, 39.4% acid resins, 10.3% neutral resins, 21.4% polycyclic aromatics, 1.5% bicyclic aromatics, 1.5% monocyclic aromatics, and 6.6% saturates [77]. Its mass C/H/N/S composition was 82.3/9.1/1.6/5.8.

Asphaltene/PP composites were produced in the melt using a twin-rotor mixer Polydrive (Haake, Vreden, Germany) equipped with sigma-shaped rotors. Polypropylene and asphaltenes were mixed in mass ratios of 95/5, 90/10, 80/20, and 70/30 at a rotor speed of 30 rpm and 180 °C for 30 min.

For investigating the strength properties and structure of asphaltene/polypropylene composites, their films were formed on an HLCL-1000 laminator (Cheminstruments, Fairfield, OH, USA) between two layers of a siliconized antiadhesive film based on polyimide at 180 °C. The thickness of the formed films was 330 ± 10 μm.

### 2.2. Methods

The morphology of composite films in a thin layer was assessed using the microphotographs obtained by optical microscopy using an MBI-1 microscope (LOMO, Saint Petersburg, Russia) and a digital IMX226 camera (Sony, Tokyo, Japan) with a resolution of 12 MP and a sensor size of 1/1.7″.

A LESCO C636 device (American Ultraviolet West, Torrance, CA, USA) was used as a source of UV radiation for the accelerated aging of composite films. The film samples were placed on a moving conveyor belt that shifted them under a UV source of 6 lamps, each with a rated power of 300 watts per inch (WPI). The duration of each film in the device was 4 min, whereas the film was exposed directly to UV radiation for 40 s. A UV lamp rated at 300 WPI generates UV radiation with a specific power ranging from 1 to 4 W/cm^2^ [78]. Thus, the average UV radiation dose for each film sample from 6 lamps was about 600 J/cm^2^. Since intense UV radiation caused heating of the films and distortion of their shapes, they were re-formed after irradiation on the laminator under the same conditions as the original non-irradiated films.

X-ray diffraction (XRD) patterns of composite films before and after UV irradiation were obtained using a diffractometer Rotaflex RU-200 (Rigaku, Tokyo, Japan) with a rotating copper anode and a horizontal wide-angle goniometer D/Max-B (Rigaku). The measurement range of diffraction angles 2*θ* was from 3 to 90 degrees. The measurement was carried out in the continuous scanning mode at a rate of 4 deg·min^−1^ and a step of 0.04 degrees. The wavelength of monochromatic radiation was 1.5405 Å.

The rheological properties of composite melts before and after UV irradiation were studied at the temperature of their blending and forming (180 °C) on a DHR-2 rotary rheometer (TA Instruments, New Castle, DE, USA) with a cone–plate unit (with a plate diameter of 25 mm and a 2° angle between the cone generatrix and the plate). The dependencies of the viscosity (*η*) on the shear stress (*σ*) were obtained by a stepwise increase in the shear rate from 0.001 to 100 s^−1^. The frequency dependencies of the storage (*G*′) and loss (*G*″) moduli were measured in the region of linear viscoelasticity by varying the angular frequency (*ω*) from 0.0628 to 628 rad·s^−1^ at a strain amplitude (*γ*) of 0.1%. Large amplitude oscillatory shear tests were performed at an angular frequency of 6.28 rad·s^−1^ by step-like increasing the strain amplitude from 0.01–0.1% to 1000% and computing the nonlinear storage and loss moduli using the first harmonic of the shear stress [79]. The equations for calculating other rheological characteristics are provided elsewhere [80,81]. The standard deviations of their estimation did not exceed 5%.

Differential scanning calorimetry (DSC) was used to evaluate the effect of asphaltenes on crystallinity and phase transition temperatures. The asphaltene/PP composite films before and after UV irradiation were studied with an MDSC 2920 instrument (TA Instruments) in an argon atmosphere at a temperature rise rate of 10 deg·min^−1^ in the heating mode from 25 °C to 210 °C and reverse cooling at the same rate.

To measure the tensile strength and Young’s modulus, we used specimens from polypropylene and its composites with asphaltenes before and after UV irradiation, which were cut from the corresponding composite films in the form of rectangles with a width of 6–9 mm and a length of 6–7 cm. The measurements were done with a TT-1100 tensile testing machine (ChemInstruments, Fairfield, OH, USA) at 25 °C and a stretching rate of 3.8 cm/min.

## 3. Results and Discussion

### 3.1. Structure of Asphaltene/Polypropylene Composites

The structure of asphaltenes in their blends with polypropylene (particle size and the presence of aggregates) was evaluated using transmission optical microscopy. Polypropylene filled with asphaltenes and original asphaltenes were used as thin films (in the case of asphaltenes, placed between two cover glasses).

**Figure 1:**
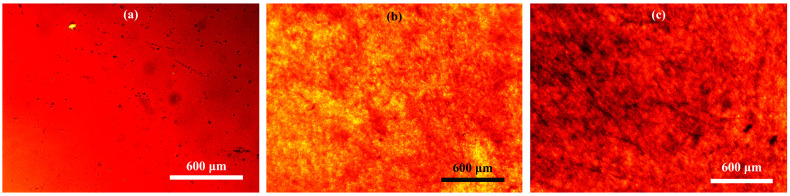
Microphotographs of films of asphaltenes (**a**) and polypropylene composites containing 10% (**b**) or 20% (**c**) of asphaltenes at 25 °C.

The microphotograph of the original asphaltenes in the glassy state shows solid particles in the asphaltene media (Figure 1a), most likely carbenes and carboids [82,83], insoluble in most organic liquids [84,85]. When asphaltenes are added to colorless polypropylene, the samples of the resulting composites acquire a yellow-brown color in a thin layer, which indicates at least a partial dissolution of asphaltenes in the polymer medium. Undissolved asphaltene particles in polypropylene have a size of about 5 μm, demonstrating good dispersibility of asphaltenes in the polypropylene matrix (Figure 1b,c). In this case, large aggregates of asphaltenes are not traced even at high degrees of asphaltene filling.

Thus, it can be concluded that the use of an aliphatic polymer as a matrix whose structure is complementary to the aliphatic peripheral groups of asphaltenes leads to good interaction of asphaltenes with the polymer and, accordingly, to high dispersibility of asphaltenes with the absence of coarse asphaltene aggregates in the polymer medium.

**Figure 2:**
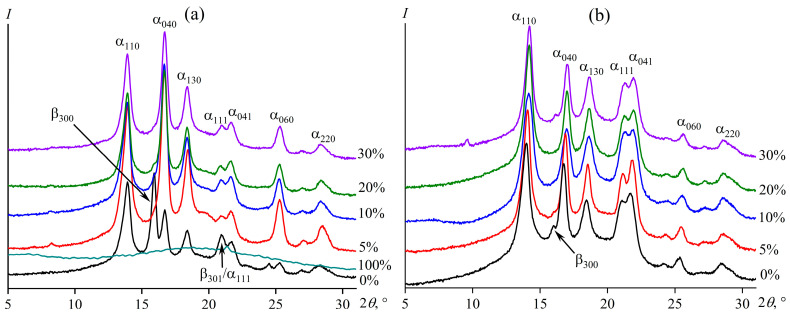
X-ray diffraction patterns of polypropylene, pure asphaltenes, and their composites before (**a**) and after (**b**) UV exposure. The asphaltene mass content is indicated near the curves.

Both asphaltenes and UV radiation can affect the crystal structure of polypropylene. Figure 2a shows X-ray diffraction patterns of pure polypropylene, asphaltenes, and their composites before UV exposure. In the diffraction pattern of asphaltenes, an extensive blurred halo can be distinguished in the 2*θ* region of 20°, while the diffractogram of polypropylene has many sharp reflections indicating its crystallinity. Polypropylene can exist in α (monoclinic), β (trigonal), γ (triclinic), and δ (smectic) crystalline forms [86,87,88]. In our case, the initial film from polypropylene has reflections corresponding to both the α phase (13.9° (α_110_), 16.7° (α_040_), 18.4° (α_130_), 21.7° (α_041_), and 25.3° (α_060_)) and the β phase (15.9° (β_300_)); reflections corresponding to α_111_ and β_301_ overlap at 20.9°. In the first approximation, the total crystallinity degree of PP can be calculated as the ratio of the sum of the reflection areas (except for the area of the amorphous halo) to the total area under the XRD pattern [89]. For the original polypropylene, the degree of crystallinity is 44.1%.

The addition of even 5% of asphaltenes leads to the disappearance of the specific reflection corresponding to the β phase (shown by the arrow in Figure 2a), while those of the α crystals remain and even become more pronounced. Calculation of the crystallinity degree shows that the asphaltenes increase the crystallinity degree of composites due to the rise in the crystallinity of polypropylene (Table 1). The higher the concentration of asphaltenes is, the higher the degree of crystallinity. Here, 30% of asphaltenes increase the crystallinity by 1.6 times from 44% to 72%. Thus, the asphaltenes act as nucleators (as shown earlier for paraffin wax [90,91], polyethylene [56], and polypropylene [60]), facilitating the crystallization of polymer chains and promoting the formation of monoclinic crystals.

The intense effect of UV on polypropylene leads to its heating, melting of crystalline regions, and partial destruction of macromolecules. As a result, polypropylene becomes brittle, and its crystallization with the formation of the β phase is suppressed sharply: the reflection at 15.9° becomes almost invisible against the background of the α phase reflections (Figure 2b). The crystallinity degree of polypropylene also reduced from 44% to 39% (Table 1). The addition of asphaltenes does not qualitatively change the diffraction pattern of composites after UV in any noticeable way (Figure 2b), although the crystallinity degree of polypropylene nevertheless increases from 39% to 62% with the addition of 30% of asphaltenes due to their nucleating effect (Table 1). Meanwhile, films containing asphaltenes do not become brittle after UV exposure, which may indicate the anti-aging influence of asphaltenes and less damage of macromolecular chains in their presence. The rupture of macromolecules with the formation of reactive free radicals should substantially affect their rheological properties, which are extremely sensitive to the molecular weight, three-dimensional crosslinks, or branching of macromolecules [92,93,94]. Let us consider the rheology of the polypropylene melt containing asphaltenes before and after UV aging.

### 3.2. Rheological Properties of Asphaltene/Polypropylene Composites

**Figure 3:**
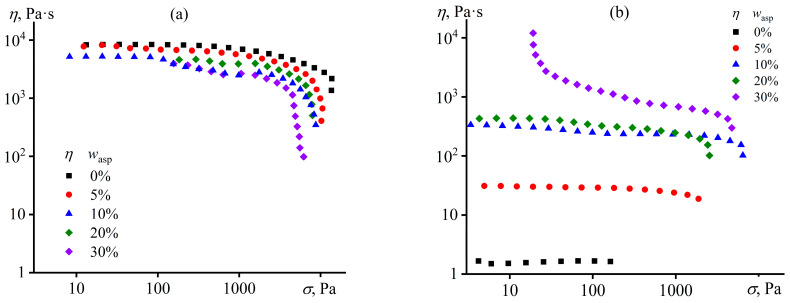
Dependencies of viscosity on shear stress at 180 °C for asphaltene/polypropylene composites before (**a**) and after (**b**) UV treatment. The asphaltene mass fraction is indicated in the legends.

The melt of the original polypropylene is a non-Newtonian fluid whose viscosity decreases at high shear stresses due to disentanglement of macromolecules, orientation of macromolecular chains, and reduction in the entanglement density (Figure 3a). Asphaltenes reduce the viscosity, which may be due to their partial solubility as lower-molecular-weight substances within the higher-molecular-weight polypropylene. Our rough estimation of the solubility of these asphaltenes in polypropylene by laser interferometry shows that it is about 7.5% at temperatures above 170 °C. In addition, the viscosity of the asphaltene/PP composites is significantly lower than that of the original polymer at high shear stresses, possibly due to the slip at the polymer–asphaltenes interface because of their nonpolar natures and, as a result, a low interfacial adhesion.

UV treatment causes a significant reduction in the viscosity of all samples (Figure 3b). The viscosity of pure polypropylene decreased by 5000 times, which can only be associated with a drop in molecular weight due to the breakage of polymer chains. In general, the viscosity of a polymer melt is proportional to the molecular weight [95]:

*η* = *KM^n^*, (1)

where *K* is the fitting parameter, and *n* is the exponent, typically 3.4 for polymers and 1.0 for oligomers. If we assume that destruction of the chains does not lead to oligomerization of macromolecules (i.e., *n* is 3.4), then the molecular weight drops by approximately 12 times due to UV exposure according to the reduction in the viscosity of pure PP. Since its initial molecular weight is 2.3 × 10^5^ g/mol, it decreases to 0.19 × 10^5^ g/mol. Usually, a polymer in bulk becomes flexible when its molecular weight is 8 to 10 times higher than the molecular weight between entanglements [96], which is about 6300 g/mol for PP [97]. In our case, the ratio between the UV-treated PP molecular weight and the entanglement one is only three, explaining the brittleness of polypropylene films after UV exposure.

Asphaltenes raise the viscosity of the UV-treated melt from 10 to 1000 times when their content increases from 5% to 30%, respectively (Figure 3b). At the same time, asphaltenes reduce the viscosity without UV exposure (Figure 3a). Thus, asphaltenes increase the viscosity of the UV-treated composites due to preventing the breaking of macromolecular chains under the influence of UV rays. Most likely, asphaltenes absorb UV radiation on the surface of the polymer composite film, preventing it from penetrating deep into the material and destroying macromolecular chains. Nevertheless, some destruction of the chains still happens even at a 30% mass fraction of asphaltenes. However, the drop in the viscosity occurs only 3.9 times rather than 5000 times as it was without asphaltenes. In the first approximation, a decrease in the viscosity by 3.9 times corresponds to a reduction in the polymer molecular weight by 1.5 times, according to Equation (1). Thus, 30% asphaltenes suppress the decrease in polypropylene viscosity by 1300 times. When converted, this suppression equals a reduction in molecular weight drop by eight times or a lowering in the destruction of polymer chains by approximately seven times. The same calculation for 5%, 10%, and 20% of asphaltenes shows the suppression of chain destruction by 1.5, 5, and 6 times, respectively.

Meanwhile, the composite containing 30% asphaltenes acquires a yield stress of about 20 Pa after UV treatment (Figure 3b). The yield stress may indicate the presence of a spatial percolation network from asphaltene particles with a strength equal to the yield stress [98,99]. The composite does not flow at shear stresses below the yield stress, whereas a shear stress equivalent to the yield stress breaks the percolation structure and causes flow. The yield stress is repeatable upon retesting the same sample, indicating that the asphaltene structure formation is reversible, i.e., due to coagulation interactions between asphaltene particles rather than their covalent cross-linking. A similar reversible structure formation causing yield stress behavior was observed for asphaltenes added to the epoxy resin in lower concentrations, without UV exposure, and before chemical curing [54]. Meanwhile, the structuring of asphaltenes in polypropylene occurs only at their high concentration and after UV treatment. In general, the structuring of solid particles in a polymer medium becomes more substantial as their concentration increases and the molecular weight of the polymer decreases, as macromolecule entanglements suppress the formation of a gel network from the particles [100]. Epoxy oligomer molecules do not entangle, and asphaltenes cause a yield stress behavior in the epoxy medium even at a 7% mass fraction [54], but do not form a gel structure in the UV-untreated polypropylene—even at four times higher content. Therefore, the appearance of yield stress behavior for the UV-treated polypropylene containing 30% asphaltenes is likely due to a decrease in its molecular weight under the action of UV, which causes less hindrance of macromolecular entanglements to the structuring of asphaltene particles, whose concentration is high. It is even possible that the initial composite containing 30% asphaltenes before UV exposure also has yield stress, which, however, is not experimentally noticeable due to its low value and high blend viscosity. In any case, the decrease in molecular weight of polypropylene and structuring of asphaltenes with the formation of the percolation network should also affect the viscoelasticity, the study of which can provide additional information about changes in the structure and properties of composites under the influence of UV.

**Figure 4:**
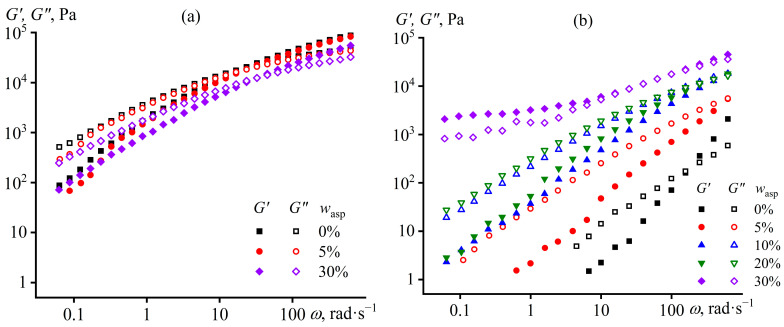
Dependencies of storage and loss moduli on the angular frequency at 180 °C for asphaltene/polypropylene composites before (**a**) and after (**b**) UV treatment. The asphaltene mass fraction is indicated in the legends.

The viscoelastic behavior of the initial polypropylene melt is typical for linear polymers (Figure 4a). The loss modulus (characterizing energy dissipation during deformation due to internal friction) exceeds the storage modulus (representing energy storage due to elasticity) at low frequencies, i.e., the melt behaves like a liquid. An increase in angular frequency leads to a growth in the moduli, and the storage modulus increases more intensively than the loss one. As a result, the storage modulus exceeds the loss modulus at high frequencies, i.e., the melt behaves like a solid, exhibiting a rubber-like behavior. The asphaltene addition into polypropylene decreases the storage and loss moduli without a qualitative change in the pattern of their frequency dependencies (Figure 4a). The decrease in viscoelasticity, as in the case of viscosity reduction, is presumably due to the partial solubility of low-molecular-weight asphaltenes within the polymer. In this case, asphaltenes act as a plasticizer for polypropylene, reducing its viscoelasticity.

When the original polypropylene is exposed to UV, a multiple decrease in its storage and loss moduli occurs (Figure 4b). At the same time, the melt’s behavior remains qualitatively the same: liquid-like at low frequencies (since *G*″ > *G*′) and solid-like at high ones (*G*″ < *G*′). Thus, UV exposure reduces the molecular weight of polypropylene, decreasing its moduli in a similar way to viscosity reduction (see Figure 3). However, UV radiation does not cause chain cross-linking, as the storage modulus would be independent of the angular frequency otherwise and exceed the loss modulus due to gel network formation [101,102].

The asphaltene addition prevents the aging of polypropylene since the storage and loss moduli of the composites decrease less intensively under the influence of UV (Figure 4b). In this case, their behavior is typical for polymer melts (*G*″ > *G*′ at low *ω*) when the asphaltene content is 5–20%. More asphaltenes result in higher moduli and, thus, less destruction of polypropylene. However, in the case of 30% asphaltene content, the moduli change very little at low frequencies, and the storage modulus exceeds the loss one, i.e., the composite exhibits gel-like behavior. This behavior is likely due to the appearance of a spatial network, possibly caused by structuring of asphaltene particles at high concentrations. When the angular frequency increases above 10 rad/s, the storage and loss moduli start to rise, perhaps due to the viscoelasticity of the polypropylene matrix. In other words, the viscoelasticity of the composite containing 30% asphaltenes is determined by the percolation network of asphaltenes at low frequencies and macromolecular entanglements at high ones. Note that without UV exposure, the composite containing 30% asphaltenes does not exhibit gel-like behavior at low frequencies. This means that UV radiation induces structure formation from asphaltenes, resulting in gel-like and yield stress behavior, but only at a 30% content.

**Figure 5:**
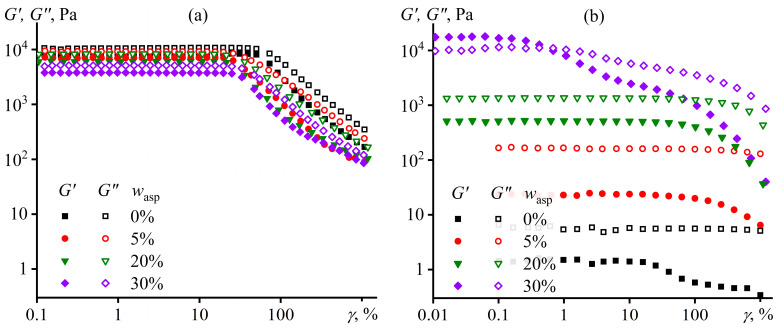
Dependencies of storage and loss moduli on the strain amplitude at 180 °C for asphaltene/polypropylene composites before (**a**) and after (**b**) UV treatment. The asphaltene mass fraction is indicated in the legends.

Additional confirmation of the structure formation comes from amplitude dependencies of storage and loss moduli (Figure 5). The initial polypropylene melt maintains linearity of mechanical properties up to a strain amplitude of about 50% (Figure 5a), similar to regular polymer solutions and melts [103,104]. The asphaltene addition slightly decreases the moduli and reduces the linear viscoelastic region to 33% at 30% asphaltene mass fraction. UV treatment acts much stronger and differently, depending on the asphaltene content (Figure 5b). When the asphaltene mass fraction changes from 0% to 20%, UV sharply reduces the moduli, especially the storage one, but does not noticeably change the length of the linear viscoelastic region. In addition, the moduli become less dependent on the strain amplitude in the nonlinear region, possibly because of the smaller number of entanglements per macromolecule due to the decline in the polymer molecular weight. The situation is different at 30% asphaltene content when UV exposure elevates the moduli but sharply reduces the linear viscoelastic region to a strain amplitude of about 0.2%. Possibly, this strain amplitude destroys the brittle network from asphaltene particles, causing the moduli to decrease to values characteristic of an unfilled polymer matrix and switching the behavior of the asphaltene/polypropylene blend from solid-like (*G*′ > *G*″) to liquid-like (*G*′ < *G*″). A further increase of the strain amplitude leads to subsequent reduction of the storage and loss moduli due to the disentanglement of macromolecules and decrease in their entanglement density as for a regular polymer melt.

Thus, according to rheological data, UV radiation destroys polypropylene chains, whereas the asphaltenes suppress both the effect of UV radiation and the destruction of chains, although incompletely. Both chain destruction and asphaltene content can affect the melting point of polypropylene, similar to how they change its degree of crystallinity according to XRD data. Let us consider the melting and crystallization of composites in more detail.

### 3.3. Calorimetry of Asphaltene/Polypropylene Composites

**Figure 6:**
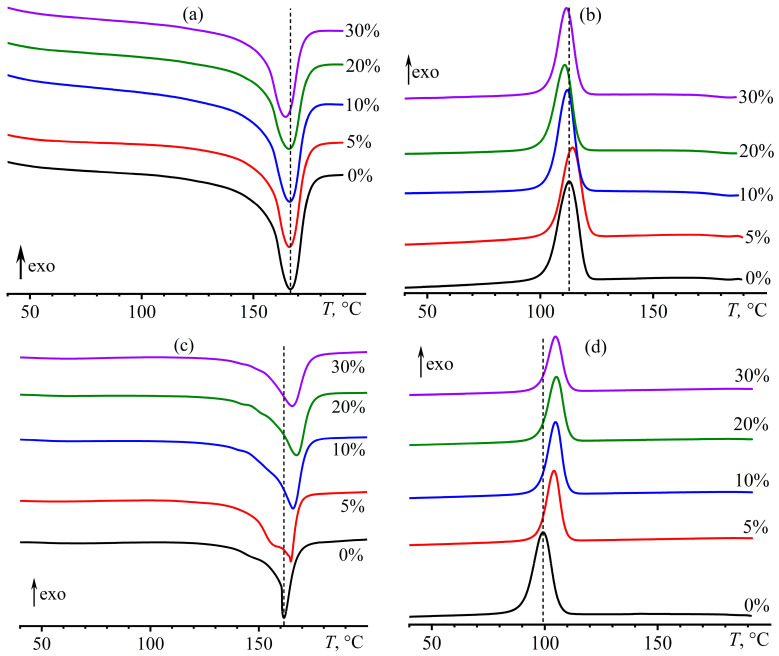
DSC thermograms of asphaltene/polypropylene composites before (**a**,**b**) and after (**c**,**d**) UV aging in heating (**a**,**c**) and cooling (**b**,**d**) modes. The asphaltene mass fractions are near the curves. Vertical dashed lines indicate the melting or crystallization temperature of pure polypropylene.

The addition of asphaltenes insignificantly affects the melting of polypropylene: the endothermic peak shifts slightly to the region of low temperatures, and its area, which is related to the degree of crystallinity of polypropylene, decreases (Figure 6a). A decrease in the melting point may be associated with a size reduction of polypropylene crystals or the appearance of defects in them. Asphaltene particles act as nucleation centers, forming numerous crystals that cannot grow to bigger sizes because of their large amount and mutual spatial restrictions. In addition, it cannot be excluded that the asphaltenes may contaminate polypropylene crystals due to their partial solubility. At the same time, the higher the asphaltene content is, the lower the melting temperature, decreasing from 166.6 °C to 164.3 °C. The melting temperatures and enthalpies for the composites are presented in Table 2 (*T*_m_ and Δ*H*_m_).

A small amount of asphaltenes (5%) increases the crystallization temperature (Figure 6b), most likely because asphaltene particles are crystallization nucleators. A further increase in the content of asphaltenes reduces the crystallization temperature to the initial level (*T*_cr_, Table 2). This decrease may indicate that asphaltenes have a plasticizing impact on polypropylene. When the asphaltene content increases, the overall thermal effect of crystallization of the composites (Δ*H*_cr_) decreases due to a reduction in the proportion of crystallizing polymer. In our case, the average melting/crystallization enthalpy of pure polypropylene is 118.1 J/g (Δ*H*_PP_), while the hypothetical melting enthalpy of this polymer with a 100% degree of crystallinity is 209 J/g [105]. Thus, the crystallinity degree of polypropylene is about 56.5% according to DSC, which is slightly higher than according to XRD (44.1%, Table 1) due to different evaluation methods.

A nominal degree of crystallinity is convenient for the comparative evaluation of the asphaltenes’ effect on the crystallinity of polypropylene:

(2) $$\mathrm{NDC}=\frac{\left(\Delta H_{m}+\Delta H_{\mathrm{cr}}\right)}{\left(100-w_{\mathrm{asp}}\right)\left(2\Delta H_{\mathrm{PP}}\right)}\cdot 100\%$$

where *w*_asp_ is the asphaltene content. NDC calculation shows that the crystallinity degree of polypropylene declines by 7–10% of the original value (Table 2). This decrease contradicts the XRD data, according to which the degree of crystallinity increased (Table 1). The explanation of the contradiction lies in the change in the phase composition of polypropylene crystals. The crystalline part of the original polymer consists of α and β phases (see Figure 2 and its discussion), whereas the addition of asphaltenes or UV exposure suppresses the formation of the β crystals, leading to the dominance of the α ones. In turn, the α and β crystals have different diffraction patterns: the α phase has seven reflections in the region of 5–30° (Figure 2), while the β one has only two in the same region. As a result, at the same degree of crystallinity, the area of reflections of the α phase is higher than that of the β one. Asphaltenes significantly reduce the content of the β crystals, thereby increasing the reflection areas and the degree of crystallinity compared to the original polypropylene sample containing both α and β crystals in comparable amounts. Thus, the estimation of the crystallinity degree by DSC data is closer to the truth, although it is also approximate since the melting enthalpies of pure α and β phases are different: 177 J/g versus 168.5 J/g, respectively [106]. Since the α crystals have a higher melting enthalpy and their proportion increases with the addition of asphaltenes, a reduction in the melting enthalpy of polypropylene clearly indicates a slight decrease in its crystallinity degree under the influence of asphaltenes.

The melting point of pure polypropylene reduces by 5 °C after UV treatment (Table 2), most likely due to a decrease in its molecular weight and/or in the size of the crystals. In this case, the nominal degree of crystallinity remains at the same level. However, since UV radiation causes a decrease in the fraction of the β phase having a lower melting enthalpy, a slight reduction in the crystallinity degree may in fact occur, but by no more than 5% of the initial value, i.e., no more than the difference in the melting enthalpies of α and β phases. The asphaltene addition causes a shift of the melting peak toward higher temperatures (Figure 6c), especially in the case of 20% asphaltenes when the melting temperature exceeds that of the same composite before its UV treatment. The increase in melting temperature compared to UV-treated asphaltene-free PP is associated with the suppression of the destruction of macromolecular chains by asphaltenes. At the same time, the crystallization temperature of pure polypropylene after UV exposure decreases, most likely due to a reduction in its molecular weight. Asphaltenes increase the crystallization temperature to about the same level regardless of their content (Figure 6d), most likely due to their action as nucleators. In addition, asphaltenes reduce the nominal degree of crystallinity by 4–11% of the value characteristic of the UV-treated pure PP, most likely due to their plasticizing effect.

Thus, although asphaltenes prevent the destruction of macromolecular chains, they reduce the crystallinity degree of polypropylene much more than UV exposure. In turn, the degree of crystallinity may affect the mechanical strength of the polymer, similar to the decrease in molecular weight under UV.

### 3.4. Strength Properties of Asphaltene/Polypropylene Composites

The asphaltene addition into the polymer aims to suppress the degradation of mechanical properties under the influence of UV. In our case, the tensile strength (*σ*_0_) and elastic modulus (*E*_0_) of polypropylene composites decrease with an increase in the mass fraction of asphaltenes (Table 3), most likely due to their plasticizing effect. The tensile strength sharply drops at an asphaltene content of 30%, possibly due to the formation of asphaltene aggregates acting as stress concentrators. Nevertheless, it can be considered that the composites containing up to 10% asphaltenes are not inferior in strength to the original polypropylene, as their strength characteristics vary within the measurement error.

UV treatment causes a significant decrease in the mechanical properties of all samples. The tensile strength and elastic modulus of polypropylene drop by more than 15 and 40 times, respectively (*σ*_UV_ and *E*_UV_, Table 3), most likely due to a decrease in molecular weight and embrittlement under the influence of UV. The addition of asphaltenes causes a gradual increase in strength values, which is due to the suppression of molecular chain destruction. The highest strength after UV aging is observed for the composite containing 20% asphaltenes, while the most increased stiffness is for the samples containing 20–30% asphaltenes. Thus, the optimal mass fraction of asphaltenes in polypropylene to improve its UV resistance is 20%. This concentration of asphaltenes makes it possible to limit the reduction in strength and stiffness of polypropylene, which decreases only by two times from the original values. The asphaltene mass fraction of 30% leads to a decrease in the strength of the UV-treated composite, which may be due to its low strength even before UV exposure.

The final issue is understanding why asphaltenes act as stabilizers against UV degradation of polypropylene. In general, there are two main possible mechanisms for UV absorbers: surface screening to control the amount of radiation that reaches the polymer and interior free-radical scavenging to inhibit chemical reactions initiated by the absorption of the radiation [107,108]. One can assume that asphaltenes can act by two mechanisms simultaneously due to their complex multi-component composition. The surface blocking is realized due to the high absorption capability of asphaltenes as polycyclic aromatic hydrocarbons towards UV radiation [61]. Asphaltenes prevent UV radiation from penetrating deep into the material, absorbing and dissipating it as heat, which is visually reflected in their black color and strong coloring ability towards polymers or aromatic solvents. However, asphaltenes can also operate as radical scavengers, excited-state quenchers, and hydroperoxide decomposers due to their aromatic ring character (similar to carbon black [109]) and vanadium complexes [110], which can potentially act as comparable metal complexes found in sulfur-containing compounds [111].

## 4. Conclusions

In this study, asphaltenes were for the first time used as a UV stabilizer against the aging of polymers. It involved the analysis of rheological, thermophysical, and strength properties of polypropylene composite films before and after intense UV aging, which revealed the following conclusions:

- Asphaltenes act as plasticizers for polypropylene, slightly reducing its viscosity, melting point, crystallinity, tensile strength, and stiffness, most likely due to their limited solubility in the polymer medium and low molecular weight;
- UV exposure dramatically reduces the viscosity and strength of polypropylene because of numerous breaks in the polymer chains, although their crystallinity is maintained, making polypropylene films brittle due to a drop in molecular weight and its approach to the molecular weight between entanglements;
- UV radiation and asphaltenes lead to the predominant formation of monoclinic crystals (α phase) and a decrease in the content of trigonal ones (β), which may be due to the break of chains and the action of asphaltenes as crystallization nucleators;
- Asphaltenes effectively absorb UV radiation, suppressing the destruction of polymer chains several times and vastly increasing the strength and rigidity of aged films compared to asphaltene-free polypropylene after UV exposure;
- Although 30% of asphaltenes reduce the destruction of the polymer by seven times, they also decrease the strength of the polymer by half, possibly due to aggregation. Therefore, the optimal mass fraction of asphaltenes is 20%, which reduces the destruction of macromolecular chains by six times and gives the best strength of the polymer films after UV aging.

The advantages of asphaltenes as a UV stabilizer are their low cost and rational utilization within polypropylene. The disadvantages are the high concentration of asphaltenes (although this can be considered an advantage due to their lower worth compared to polypropylene), the black color of the resulting blends, and their impossibility of use in contact with food and human skin due to the potential carcinogenicity and toxicity of asphaltenes as polycyclic aromatic hydrocarbons partially dissolved within the polymer matrix. Asphaltene-containing polymer composites should be used cautiously and in limited areas that exclude possible health hazards. In addition, a typical concentration of a UV stabilizer is usually fractions of a mass percent, while the adequate content of asphaltenes is tens of percent, making an asphaltene/polymer blend an asphaltene-filled polymer composite rather than an asphaltene-modified polymer. The limitations of this study include the use of accelerated UV aging, lack of consideration of the kinetics of UV destruction of polymer chains, and indirect evaluation of changes in their molecular weight. Further work should aim at overcoming these limitations and furthering the research on asphaltenes as UV stabilizers to other polymers.

## Figures and Tables

**Table 1:** The crystallinity degree of polypropylene composites containing different mass fractions of asphaltenes according to XRD data before and after UV irradiation.

*w*_asp_, wt%	Before UV	After UV
0	44.1% (44.1%) ^1^	39.1% (39.1%)
5	52.3% (55.1%)	43.0% (45.3%)
10	48.5% (53.9%)	43.4% (48.2%)
20	52.5% (65.6%)	41.3% (51.6%)
30	50.4% (72.0%)	43.6% (62.2%)

^1^ The data in parentheses show the crystallinity degree of polypropylene alone after recalculation accounting for the content of asphaltenes in the composite.

**Table 2:** The melting and crystallization of asphaltene/polypropylene composites before and after UV treatment.

*w*_asp_, wt%	UV	*T*_m_, °C	Δ*H*_m_, J/g	*T*_cr_, °C	Δ*H*_cr_, J/g	NDC, %
0	before	166.6	114.9	112.7	121.3	100
5	before	166.1	103.4	114.2	106.2	93.4
10	before	166.2	87.7	112.0	105.0	90.6
20	before	166.0	81.3	110.9	92.5	92.0
30	before	164.3	67.2	111.6	87.4	93.5
0	after	161.5	75.4	99.3	159.2	99.3
5	after	164.8	92.2	104.1	105.2	88.0
10	after	165.8	85.5	104.7	104.4	89.3
20	after	167.5	81.8	105.1	98.1	95.2
30	after	165.4	66.8	104.7	81.9	89.9

**Table 3:** The tensile strength and elastic modulus of asphaltene/polypropylene composites before and after UV treatment.

*w*_asp_, wt%	*σ*_0_, MPa	*σ*_UV_, MPa	*E*_0_, MPa	*E*_UV_, MPa
0	28 ± 2	1.8 ± 0.4	520 ± 80	13 ± 14
5	28 ± 4	4.2 ± 1.1	470 ± 100	50 ± 15
10	26 ± 4	6.1 ± 1.5	410 ± 130	50 ± 50
20	22 ± 5	9.9 ± 2.3	250 ± 150	100 ± 50
30	12 ± 2	6.5 ± 1.6	380 ± 70	120 ± 50

## Data Availability

The data presented in this study are available on request from the corresponding author.
